# Limitation of Laryngeal Mask Airway Usage in Open-Neck Procedure

**Published:** 2019-07

**Authors:** Fatma Caylakli

**Affiliations:** *Department of Otorhinolaryngology Head and Neck Surgery, Baskent University School of Medicine, Ankara, Turkey.*

**Keywords:** Laryngeal mask airway, Open, Neck

 I read the manuscript entitled “Case Report of An Open Neck Procedure Complication Associated with Laryngeal Mask Airway Use” by Subramaniam T et al. This is a valuable manuscript describing iatrogenic complications during level-2 cervical lymph node biopsy, in which the airway is managed with laryngeal mask airway (LMA). During the procedure, the surgeon realized that the mass was actually the LMA and that the pharyngeal wall had been dissected, resulting in an iatrogenic pharyngeal tear.

 LMA is a safe method to establish airway control during general anesthesia; however, it is controversial during open-neck procedures. In particular, during lymph node biopsy and branchial cyst excision procedures, the surgeon can feel the cuff of the LMA as a mass, and complications can occur during surgery. Besides these complications, LMA is not appropriate during lymph node biopsy procedures, particularly for level-2 cervical lymph nodes. During LMA usage for anesthesia, the natural position of the neck disappears and it is not possible to palpate the lymph node. Even if the surgeon marks the place of the lymph node before the operation, after LMA usage the surgeon is not able to palpate the lymph node accurately, and may even lose the lymph node. Therefore, during open-neck procedures, endotracheal intubation is a more appropriate technique than LMA usage.

